# The Role of Marine Organic Extract in Bone Regeneration: A Pilot Study

**DOI:** 10.1155/2020/2925879

**Published:** 2020-02-17

**Authors:** João César Zielak, Ivana Vendramini, Paola Fernanda Cotait de Lucas Corso, Leonardo Luiz Muller, Viviane Rozeira Crivellaro, Sharukh Soli Khajotia, Fernando Luis Esteban Florez, Rafaela Scariot, Mohammed Elsalanty, Tatiana Miranda Deliberador, Carmen Lucia Mueller Storrer

**Affiliations:** ^1^School of Health Sciences, Universidade Positivo, Rua Professor Pedro Viriato Parigot de Souza, 5300 Curitiba, Paraná 81280-330, Brazil; ^2^The University of Oklahoma Health Sciences Center, Department of Restorative Sciences, Division of Dental Biomaterials, College of Dentistry, 1201 N. Stonewall Avenue, Oklahoma City, Oklahoma 73117, USA; ^3^Augusta University, Department of Oral Biology and Diagnostic Sciences, Dental College of Georgia, Augusta, Georgia 30912, USA

## Abstract

Novel biomaterials capable of accelerating the healing process of skeletal tissues are urgently needed in dentistry. The present in vivo study assessed the osteoconductive and osteoinductive properties of experimental biphasic bioceramics (HA-TCP) modified or not by a nacre extract (marine organic extract, MOE) in a sheep model. Fabrication of MOE involved mixing ground nacre (0.05 g, particle sizes < 0.1 mm) with glacial ethanoic acid (5 mL, pH 7) for 72 hours using external magnetic stirring (25°C). Nonreactive carriers (sterile polythene tubes; 3/animal, radius: 2.5 mm, length: 10.0 mm) pertaining to the control (empty) or experimental groups (HA-TCP or MOE-modified HA-TCP) were implanted intramuscularly into the abdominal segment of the torso in sheep (*n* = 8, age: 2 years, weight: 45 kg). Euthanization of animals was performed at 3 and 6 months after surgery. Tissues harvested were subjected to macroscopic and radiographic assessments. Specimens were then stained for histological analysis. Both control and experimental animals were capable of inducing the neoformation of fibrous connective tissue at both time points where superior amounts of tissue formation and mineralization were detected for experimental groups (unaltered (at 3 and 6 mos) and MOE-modified HA-TCP (at 3 mos)). Histological results, however, revealed that mature bone formation was only observed for specimens fabricated with MOE-modified HA-TCP in a time-dependent manner. The present study has successfully demonstrated the in vivo utility of experimental biphasic bioceramics modified by MOE in an ectopic grafting sheep model. Promising osteoconductive and osteoinductive properties must be further developed and confirmed by subsequent research.

## 1. Introduction

It is estimated that billions of dollars are spent every year all over the world for the placement of bone defect repairs [1, 2]. In this critical context, it becomes obvious that novel implantable biomaterials capable of accelerating the natural healing of skeletal tissues are urgently needed. Important scientific and technological breakthroughs in cellular biology, in situ engineering of tissues and biomaterials development [3, 4], have resulted in the attainment of novel materials and surgical techniques that, combined, are capable of promoting in situ growth of bone tissues while displaying unprecedented levels of control and selectivity. According to previous reports [5, 6], there are many shortcomings associated with the utilization of homografts and xenografts that include limited bioavailability, tissue rejection, viral or prion contamination, and the necessity for the utilization of immunosuppressant drugs [7, 8].

Autografts are typically regarded as the gold standard for bone repair because of their high levels of histocompatibility and the presence of a 3-dimensional porous matrix containing essential osteoconductive components (i.e., bone morphogenic proteins, growth factors, osteoprogenitor cells) [9]. These types of grafts involve harvesting bone (i.e., iliac, mandibular ramus, chin) and expensive surgical procedures and have been previously correlated with surgical problems such as morbidity of the donor site, cicatrix, hemorrhage, inflammation, bacterial contamination, and chronic pain [9–12]. Synthetic bone substitutes (also known as alloplastic bone repairs) are commonly utilized as alternatives to autografts because they are readily available and they eliminate the need for a donor site [13]. Despite these promising properties, previous studies have demonstrated that alloplastic materials, such as bioceramics and synthetic hydroxyapatites, are not able to repair critical-sized calvarial defects in animal models (rabbit and rat) [13, 14]. Extensive research efforts to overcome previously cited limitations resulted in the attainment of novel biocompatible organo-biomaterials such as natural hydroxyapatite (HA) [15, 16], *β*-tricalcium phosphate (TCP) [17], and low-temperature apatites (LTA) [18]. More recently, our laboratory has reported a pilot study describing the in vivo bone repair utility of demineralized bone matrix (DBM) modified by an experimental nacre extract (MOE) in a sheep ectopic model [19]. The findings reported therein have indicated that, under the experimental conditions tested, materials investigated were not associated with significant osteoinductive and osteogenic functionalities. Therefore, the objective of the present controlled and randomized in vivo study was to determine the in vivo osteogenic functionalities of unaltered or MOE-modified biphasic bioceramics in a sheep model using a comprehensive assessment approach based on macroscopic, radiographic, and histological analyses.

## 2. Materials and Methods

### 2.1. Institutional Animal Care and Use Committee

The Animal Use Ethics Committee of the Positivo University reviewed and approved the execution of the present randomized and controlled in vivo study (approval # CEUA 80/11).

### 2.2. Nacre Extract (MOE) Preparation

Our laboratory has previously reported the methodology used for the fabrication of the experimental MOE used in the present study [19]. A local seafood supplier provided frozen *Perna perna* (brown) mussels. After being thawed, mussels were washed (30 sec each) under copious irrigation with fresh water (25°C) and were air-dried for no less than 24 hours. Mussels' shells were then reduced into a fine powder using a pestle for bone (Kopp, Paraná, Brazil) and a Teflon planetary grinding jar (AMEF, São Paulo, Brazil). Pulverized particles of nacre (0.05 g) were then mixed with glacial ethanoic acid (1 M, pH 7.0, 5 mL, 25°C) using external magnetic stirring for 72 hours (Tongtuo, Hayward, USA). Experimental MOE was then centrifuged at 4,000 rpm (15 min, MPW-350R, MPW Med. Instruments, Warsaw, Poland) and the supernatant was carefully aspirated using a calibrated pipette.

### 2.3. Approach

Female sheep (*n* = 8, 2 yrs. of age, ≈45 kg, Table 1) were triaged and selected to participate in the present controlled and randomized in vivo study. Animals were then randomized to each experimental group using the Research Randomizer freeware (V4.0, Social Psychology Network, Pennsylvania, USA) available online (http://www.randomizer.org). Nonreactive carriers (3/animal; radius: 2.5 mm, length: 10.0 mm) containing GenPhos HA-TCP (unaltered or modified by 40 *μ*L of MOE) were then implanted intramuscularly at each animal's torso abdominal segment. Empty sterile nonreactive carriers served as the control (groups 1 and 4, *sham*). Animals of each experimental group (euthanized either 3 or 6 months) received 3 tubes (either empty or containing unaltered MOE-modified HA-TCP) as shown in Table 1. The model reported (intramuscular implantation) was selected based on its capabilities to provide environments that are mechanically unstable [20] while assessing the biocompatibility and regeneration properties of implantable organo-biomaterials [21]. The materials reported in the present study were also investigated for their osteoconductive and osteoinductive properties in critical size defects (sheep mandible). However, those results will be reported in a subsequent manuscript.

### 2.4. Surgical Procedures

Sheep were identified with earrings following common practices typically used in animal breeding. The present study was conducted with the approval of the Animal Care Committee and followed the guidelines established by European Community for the handling, care, and utilization of experimental animals (DE 86/609/CEE) [22]. Animals fasted for solid food (24 hours) and water (8 hours) prior to surgery. Preanesthesia medications (acepromazine [Acepran 1%; 0.55 mg/kg, Vetnil, Brazil] and ketamine [Dopalen; 20 mg/kg, Vetbrands, Brazil]) were intramuscularly provided to the animals. Sodium thiopental (Thiopentax, 5 mg/kg, Brazil) was used intravenously to induce animals to anesthesia. A mixture of oxygen and isoflurane (Biochimico, 3 L/min, Brazil) vaporization was used to maintain animals' sedation. Other transoperative procedures included the intramuscular injection drugs with anti-inflammatory and antibiotic actions such as Ketoprofen 10% (Biopharma Quimica e Farmaceutica Ltda, Brazil; 3 mg/kg) and Enrofloxacin 10% (Chemitec Agro-Veterinária, Brazil; 2.5 mg/kg). Surgical sites (length 20.0 cm) were located 3.0 cm cranially to the sacral promontory and 6.0 cm laterally to the vertebral column (either right or left, randomly assigned). Povidone-iodine (Rioquímica, Brazil) was used to disinfect surgical sites after trichotomy procedures in preparation for incisional procedures. Separate incisions (*n* = 3, 3.0 cm/incision, 4.0 cm aside) were performed on the epidermis and dermis to allow access to the muscular fascia (*Longissimus dorsi*) region. Individual intramuscular incisions (1.0 cm/each) were performed perpendicularly to the vertebral column to allow access for the placement of nonreactive carriers containing the materials investigated within the muscular tissue. Vicryl 5-0 absorbable (Ethicon, Brazil) was used for the deep suture of the muscle and fascia using the interrupted technique. Nonabsorbable nylon 5-0 (Shalon Fios Cirúrgicos Ltda, Brazil) was used for the suture of the external dermis using a continuous technique. Anti-inflammatory and antibiotic drugs (Ketoprofen 10% [3 mg/kg] and Enrofloxacin 10% [2.5 mg/kg], respectively) were intramuscularly administered to animals 3 and 5 days after surgery.

### 2.5. Euthanization and Fixation Procedures

Euthanization of animals was performed (at 3 or 6 months) using a previously published protocol [19]. Sodium thiopental IV (8 mg/Kg, Thiopentax, Brazil) and potassium chloride (20 mL, 19.1%, Brazil) were intramuscularly administered to allow the removal of specimens (each nonreactive carrier displayed roughly 1.0 cm of encircling tissues). Specimens were fixed in neutral buffered formalin (10%, at 4°C; Sigma-Aldrich, Brazil). Subsequently, specimens were immersed for 48 hours in formaldehyde (10%) prior to the comprehensive analyses (digital images, radiographic and histologic) performed in the present study.

### 2.6. Digital Image Assessment

Histology-ready specimens were then assessed for their macroscopic features using a semiprofessional digital camera (Canon T1i, 105 mm macro lens, Oita, Japan) and digital image analysis software (ImageJ, available online at http://imagej.nih.gov/ij/).

### 2.7. X-Ray Assessment

Specimens were individually assessed for radiopacity through a conventional exam (0.005 mSv, 0.3 seconds) performed with traditional X-ray equipment (Spectro 70x, Dabi Atlante, São Paulo, Brazil) connected to a digital sensor (RVG 5100, Kodak Company, USA).

### 2.8. Histology Assessment

Trichloroacetic acid 5% (TCA; 25°C) was used (for 40 days) to demineralize the specimens. Specimens were inserted into blocks of paraffin and were serially sliced (6 *μ*m/slice) in their longitudinal plane, as commonly reported in histological studies. Individual slices were treated with hematoxylin and eosin (HE) and Masson's trichrome (MT). An optical microscope (BX 41, Olympus Optical Company, Tokyo, Japan) coupled with a digital camera (Canon T1i, Oita, Japan) was then used to image stained slices. Digital images were then processed by a single and previously trained operator using Photoshop CS4 (Adobe Systems Inc., California, USA) and ImageJ (Freeware, available at http://imagej.nih.gov/ij/).

### 2.9. Statistical Analysis

Mean values from the macroscopic (horizontal cross-section), radiographic (radiopacity levels), and microscopic assessments (mature bone areas) were analyzed for normality using the Shapiro–Wilk test. Since a normal distribution was observed, results obtained were then statistically analyzed using Student's *t*-test to compare the efficacy of the investigated interventions among selected areas. Summary statistical analyses in the present study were performed using the Statistical Package for Social Science® (SPSS, V24.0, IBM) with a confidence level of 95%.

## 3. Results

**Figure 1:**
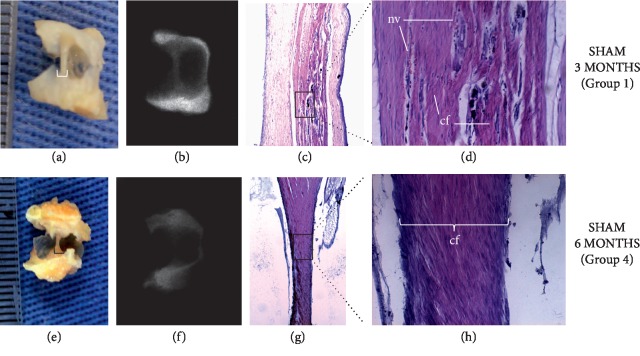
Macroscopic aspect (a, e), radiographic sections (b, f), and histological sections (c, d, g, h) with negative control or sham (tube without biomaterial). Microscopic appearance (HE, original 200x) demonstrating neovascularization (nv) and connective tissue fibers (cf).

**Figure 2:**
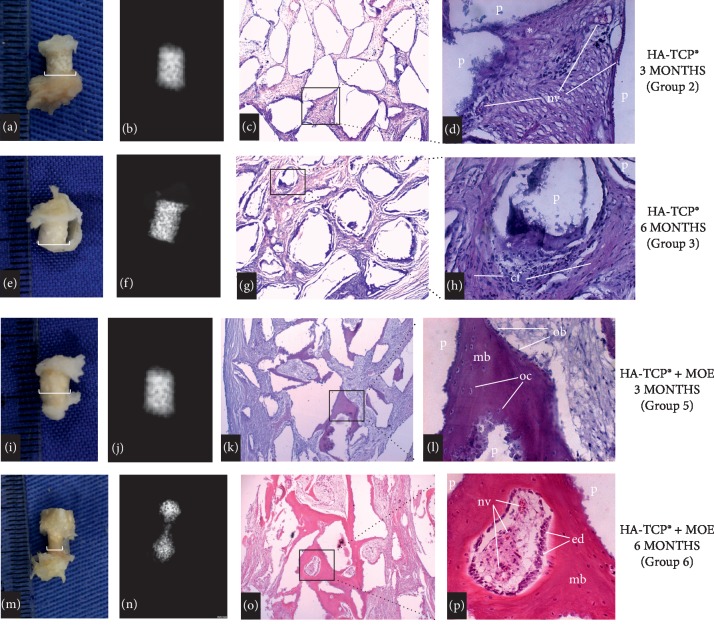
Macroscopic aspect (a, e, i, m), radiographic sections (b, f, j, n), and histological sections (c, d, g, h, k, l, o, p) with HA-TCP® with and without marine organic extract. Microscopic appearance (HE, original 200x) demonstrating particle (p) of HA-TCP, osteoid (*∗*), neovascularization (nv), connective tissue fibers (cf), mature bone (mb), osteoclast (oc), osteoblast (ob), endosteal (ed). MOE  marine organic extract.

The results of the macroscopic analysis, at 3 and 6 months, were reported in the present study in terms of representative digital images (Figures 1(a), 1(e), 2(a), 2(e), 2(i), and 2(m)) and horizontal scale bars (in terms of mm, ImageJ).

Images reported have indicated that all materials investigated were capable of promoting the growth of bone-like tissues. Specimens pertaining to either the control (1 and 4) or experimental (2, 3, and 5) groups were associated with the lowest (horizontal cross-section: 0.5 mm) and highest (horizontal cross-section: 5.0 mm) amounts of fibrotic or bone-like tissues, respectively. Specimens of experimental group 6 (GenPhos, HA-TCP + MOE, 6 mos) displayed intermediate levels of tissue growth when compared to the remaining groups investigated. The radiographic assessment (radiopaque areas) resulted in means of 0.219 ± 0.037 in 2 for groups 1 and 4, and 0.695 ± 0.005 in 2 for groups 2, 3, 5, and 6 (Figures 1(b), 1(f), 2(b), 2(f), 2(j), and 2(n)) where it is possible to observe that specimens pertaining to control groups (1 and 4) displayed the lowest radiopacity levels among all groups tested (*p* = 0.034). Histological results (at 3 and 6 mos) are displayed in Figures (1(c), 1(d), 1(g), 1(h), 2(c), 2(d), 2(g), 2(h), 2(k), 2(l), 2(o), and 2(p)). Figures 1(d) and 1(h) have shown that specimens of the control groups (either 1 or 4) were capable of promoting the growth of thin tissues that were organized as parallel bundles of loosely arranged connective fibers interspersed with blood vessels. Figures 2(d) and 2(h) illustrate the results (at 3 and 6 mos) of experimental groups containing unaltered GenPhos HA-TCP particles. In both images, it is possible to observe the presence of dense fibers, blood vessels, and unreacted HA-TCP particles. Figures 2(l) and 2(p) show the results (at 3 and 6 mos) for MOE-modified GenPhos HA-TCP. It is possible to observe in Figure 2(l) the presence of mature bone (MB), osteoclasts (OC), and osteoblasts (OB) between particles of MOE-modified GenPhos HA-TCP. Figure 2(p) illustrates the presence of neovascularization (NV) and endosteal cells (ED) within MB at six months.

## 4. Discussion

The present controlled and randomized in vivo study had the objective of comprehensively characterizing the osteoconductive and osteoinductive functionalities of unaltered or MOE-modified GenPhos HA-TCP as materials to promote the guided deposition of bone tissues. The animal model used in the present study was selected because it provides physiological and biochemical environments that, comparable to those observed in humans [23], are rapid and efficient and have been correlated with high levels of sensitivity [22] while displaying unrivaled ability to indicate the occurrence of foreign-body giant cells reactions [24].

The reason for the selection of nonreactive polymer carriers was based on results reported previously by our group [19, 25], where intramuscular implantation of tubes (identical composition and similar surgical conditions) did not generate any type of allergic or inflammatory reactions in sheep models. Figures 1(a)–1(h) illustrate the macroscopic, radiographic, and histological results for specimens pertaining to the control groups (1 and 4). Comparing the results shown in Figures 1(a) and 1(e) at 3 and 6 mos, respectively, it was possible to observe the formation of tissues displaying macroscopic features (such as color and texture) resembling those of mineralized tissues. It was also possible to observe that similar (*p* > 0.05) amounts of tissues were formed independently of the monitoring time considered (either at 3 or 6 mos). The results from the radiographic analysis shown in Figures 1(b), 1(f), 2(b), 2(f), 2(j), and 2(n) have indicated that neoformed tissues were associated with radiopacity levels that were maintained in a biomaterial presence-dependent manner. The histological results presented in Figures 1(c), 1(d), 1(g), and 1(h) have indicated the presence of cells typically associated with acute inflammation processes (such as lymphocytes and macrophages), which is considered an expected local tissue reaction in response to the implantation of tubes, and, therefore, are in good agreement with a previously published report [26]. The presence of inflammatory cells related to chronic processes, allergy, or foreign-body reactions (such as leukocytes) was not observed at neither 3 nor 6 mos, thereby further corroborating the rationale for the selection of polymer-based nonreactive carriers for the testing of the implantable organo-biomaterials investigated in the present study.

The utilization of MOE as a promoter of bone growth was supported by this material's proven biocompatibility, low antigenicity properties, and growth factors' concentration (e.g., bone morphogenic-like proteins) that are considered adequate for human utilization [27, 28]. Previous studies investigating the efficacy of bone-growth factors have demonstrated the presence of a positive and strong correlation between the occurrence of adverse effects (undesired heterotopic ossification and life-threatening inflammatory reactions) and concentrations of bone-growth factors (such as BMP, BMP-2, or rhBMP-2) [29–31]. A recent in vivo study investigating the utility of bioceramics (HA-TCP) modified or not by adipose stem cells, in an experimental canine model using dogs, has indicated based on previous scientific evidence [32] that hydroxyapatite and HA-TCP display promising osteoconductive and osteoinductive properties that were shown to trigger the repair of damaged and degenerated tissues in a contact-dependent manner [33].

The macroscopic results presented in Figures 2(a), 2(e), 2(i), and 2(m) suggest that, with the exception of group 6 (MOE-modified HA-TCP at 6 mos.), all remaining experimental groups were capable of inducing the growth of large quantities of tissues with macroscopic features that were very similar to those found in bone. The results of the radiographic analysis reported similar radiopacity levels for all the experimental groups 2, 3, 5, and 6. And although in group 6 the radiopacity area had a different morphology, the total area was similar. This difference can be explained by the histological aspects shown in this group. The results of the histological analysis reported (Figures 2(c), 2(d), 2(g), 2(h), 2(k), 2(l), 2(o), and 2(p)) have indicated that specimens pertaining to groups 2 and 5 (unaltered HA-TCP at 3 and 6 mos.) displayed the presence of similar quantities of neoformed fibro-vascular tissues and osteoid-like cells among the HA-TCP, but the formation of mature bone could be observed only when MOE was associated (groups 3 and 6), shown to display significant higher amounts of mature bone among HA-TCP particles at 6 months (*p* = 0.0003). The results reported in Figure 2(l) have also indicated the presence of bone remodeling cells (such as osteoclasts and osteoblasts). In Figure 2(p) it is possible to observe that specimens pertaining to experimental group 6 (MOE-modified HA-TCP at 6 mos) promoted the formation of larger amounts of mature and layered vascularized bone tissues with endosteal-coated spinal cavity formation, and the presence of peripherally aligned osteoblast cells and living osteocytes, which are characteristics typically found in compact bone tissues. These findings suggest that MOE-modified experimental materials displayed promising osteoinductive properties that, under the conditions and limitations of the present study, were considered of fundamental importance for the formation of new bone. Several studies have reported similar results [34–39], thereby corroborating the findings of the present study.

A study investigating the in vitro biomineralization of human osteoblasts has demonstrated that nacre is capable of attracting and activating osteoblasts [40], thereby suggesting that similar regulatory mechanisms [41] and biomineralization systems may exist in both vertebrates and mollusks [42]. Another sheep model study, investigating the interface between bone and nacre chronic implants, observed direct binding between bone and nacre implants, wherein a layer of progenitor cells was contacting, in a direct manner, the implant's surface without the presence of a fibrous intervening layer [43]. Duplat et al. [44], while investigating the in vitro osteoclastic-driven degradation of nacre (*Pinctada oyster*), have demonstrated that nacre can also modulate the differentiation of stem cells into osteoclasts that were able to resorb the material, and can be used to partially explain the results presented in Figures 2(m)–2(p) where specimens pertaining to experimental group 6 (MOE-modified HA-TCP at 6 mos) were associated with the lowest amount of tissue formation inside of the nonreactive carriers (at 6 mos).

## 5. Conclusions

The present study has successfully reported the experimental association of biphasic bioceramics modified by a marine organic extract from nacre. The comprehensive assessment approach used, suggests that experimental materials investigated (MOE-modified HA-TCP) displayed promising in vivo osteoconductive and osteoinductive functionalities in an ectopic sheep model. It is anticipated that after complete development and characterization, experimental materials reported will display active osteogenic properties. The utilization of these materials is expected to be translated into shorter healing times, materials able to heal critical-sized defects, decreased bone repair costs, and patients displaying improved quality of life. Despite the promising results reported, additional research is necessary to further develop the functionalities described and to confirm the findings of the present study.

## Figures and Tables

**Table 1:** Experimental conditions: sham  empty tube; MOE  marine organic extract; GenPhos HA-TCP®.

Groups	Type	Monitoring time	Biomaterial
1	Control	3 months	Sham
2	Experimental		GenPhos HA-TCP®
3	Experimental		GenPhos HA-TCP® + MOE

4	Control	6 months	Sham
5	Experimental		GenPhos HA-TCP®
6	Experimental		GenPhos HA-TCP® + MOE

## Data Availability

The datasets generated and analyzed during the current study are available from the corresponding author on reasonable request.

## References

[1] Amini A. R., Laurencin C. T., Nukavarapu S. P. (2012). Bone tissue engineering: recent advances and challenges. *Critical Reviews™ in Biomedical Engineering*.

[2] Zhang K., Wang S., Zhou C. (2018). Advanced smart biomaterials and constructs for hard tissue engineering and regeneration. *Bone Research*.

[3] Carbonari M., Ludtke J., dos Santos P. C. V., Amaral Carvalho N. T., Gehrke S. A. (2009). Caracterização físico-química e biológica de enxerto ósseo bovino, Bonefill, em bioensaios-parte 1. *Implantnews*.

[4] Lima A. C., Ferreira H., Reis R. L., Neves N. M. (2019). Biodegradable polymers: an update on drug delivery in bone and cartilage diseases. *Expert Opinion on Drug Delivery*.

[5] Baroli B. (2009). From natural bone grafts to tissue engineering therapeutics: brainstorming on pharmaceutical formulative requirements and challenges. *Journal of Pharmaceutical Sciences*.

[6] Dimitriou R., Jones E., McGonagle D., Giannoudis P. V. (2011). Bone regeneration: current concepts and future directions. *BMC Medicine*.

[7] Hench L. L. (1998). Biomaterials: a forecast for the future. *Biomaterials*.

[8] Danesh-Sani S. A., Engebretson S. P., Janal M. N. (2017). Histomorphometric results of different grafting materials and effect of healing time on bone maturation after sinus floor augmentation: a systematic review and meta-analysis. *Journal of Periodontal Research*.

[9] Younger E. M., Chapman M. W. (1989). Morbidity at bone graft donor sites. *Journal of Orthopaedic Trauma*.

[10] Banwart J. C., Asher M. A., Hassanein R. S. (1995). Iliac crest bone graft harvest donor site morbidity. *Spine*.

[11] Ebraheim N. A., Elgafy H., Xu R. (2001). Bone-graft harvesting from iliac and fibular donor sites: techniques and complications. *Journal of the American Academy of Orthopaedic Surgeons*.

[12] St John T. A., Vaccaro A. R, Sah A. P (2003). Physical and monetary costs associated with autogenous bone graft harvesting. *American Journal of Orthopedics (Belle Mead, N.J.)*.

[13] Mah J., Hung J., Wang J., Salih E. (2004). The efficacy of various alloplastic bone grafts on the healing of rat calvarial defects. *The European Journal of Orthodontics*.

[14] Bidic S. M. S., Calvert J. W., Marra K. (2003). Rabbit calvarial wound healing by means of seeded caprotite scaffolds. *Journal of Dental Research*.

[15] Conz M. B., Granjeiro J. M., de Almeida Soares G. (2011). Hydroxyapatite crystallinity does not affect the repair of critical size bone defects. *Journal of Applied Oral Science*.

[16] Lei X., Gao J., Xing F., Zhang Y., Ma Y., Zhang G. (2019). Comparative evaluation of the physicochemical properties of nano-hydroxyapatite/collagen and natural bone ceramic/collagen scaffolds and their osteogenesis-promoting effect on MC3T3-E1 cells. *Regenerative Biomaterials*.

[17] Horowitz R. A., Mazor Z., Foitzik C., Prasad H., Rohrer M., Palti A. (2010). *β*-tricalcium phosphate as bone substitute material: properties and clinical applications. *Journal of Osseointegration*.

[18] Šponer P., Strnadová M., Urban K. (2011). In vivo behaviour of low-temperature calcium-deficient hydroxyapatite: comparison with deproteinised bovine bone. *International Orthopaedics*.

[19] Zielak J. C., Gulin Neto D., Zielak M. A. C. (2018). In vivo regeneration functionalities of experimental organo-biomaterials containing water-soluble nacre extract. *Heliyon*.

[20] Wu G., Hunziker E. B., Zheng Y., Wismeijer D., Liu Y. (2011). Functionalization of deproteinized bovine bone with a coating-incorporated depot of BMP-2 renders the material efficiently osteoinductive and suppresses foreign-body reactivity. *Bone*.

[21] Sartoretto S. C., Uzeda M. J., Miguel F. B., Nascimento J. R., Ascoli F., Calasans-maia M. D. (2016). Sheep as an experimental model for biomaterial implant evaluation. *Acta Ortopédica Brasileira*.

[22] Lenihouannen D., Daculsi G., Saffarzadeh A. (2005). Ectopic bone formation by microporous calcium phosphate ceramic particles in sheep muscles. *Bone*.

[23] Martini L., Fini M., Giavaresi G., Giardino R. (2001). Sheep model in orthopedic research: a literature review. *Comparative Medicine*.

[24] Anderson J. M., Rodriguez A., Chang D. T. (2008). Foreign body reaction to biomaterials. *Seminars in Immunology*.

[25] da Silva R. C., Crivellaro V. R., Giovanini A. F., Scariot R., Gonzaga C. C, Zielak J. C. (2016). Radiographic and histological evaluation of ectopic application of deproteinized bovine bone matrix. *Annals of Maxillofacial Surgery*.

[26] Morais J. M., Papadimitrakopoulos F., Burgess D. J. (2010). Biomaterials/tissue interactions: possible solutions to overcome foreign body response. *The AAPS Journal*.

[27] Rousseau M., Pignatello R. (2011). Nacre, a natural biomaterial. *Biomaterials Applications for Nanomedicine*.

[28] Oliveira D. V., Silva T. S., Cordeiro O. D., Cavaco S. I., Simes D. C. (2012). Identification of proteins with potential osteogenic activity present in the water-soluble matrix proteins from *Crassostrea gigas* nacre using a proteomic approach. *The Scientific World Journal*.

[29] Zara J. N., Siu R. K., Zhang X. (2011). High doses of bone morphogenetic protein 2 induce structurally abnormal bone and inflammation in vivo. *Tissue Engineering Part A*.

[30] Carragee E. J., Hurwitz E. L., Weiner B. K. (2011). A critical review of recombinant human bone morphogenetic protein-2 trials in spinal surgery: emerging safety concerns and lessons learned. *The Spine Journal*.

[31] Lissenberg-Thunnissen S. N., de Gorter D. J. J., Sier C. F. M., Schipper I. B. (2011). Use and efficacy of bone morphogenetic proteins in fracture healing. *International Orthopaedics*.

[32] Hashemibeni B., Dehghani L., Sadeghi F. (2016). Bone repair with differentiated osteoblasts from adipose-derived stem cells in hydroxyapatite/tricalcium phosphate in vivo. *International Journal of Preventive Medicine*.

[33] Kim E.-H., Heo C. Y. (2014). Current applications of adipose-derived stem cells and their future perspectives. *World Journal of Stem Cells*.

[34] Lamghari M., Almeida M. J., Berland S. (1999). Stimulation of bone marrow cells and bone formation by nacre: in vivo and in vitro studies. *Bone*.

[35] Lopez E., Vidal B., Berland S., Camprasse S., Camprasse G., Silve C. (1992). Demonstration of the capacity of nacre to induce bone formation by human osteoblasts maintained in vitro. *Tissue and Cell*.

[36] David W. G., Hyuk-Jae K., Han-Sung J. (2015). Osteogenic potency of nacre on human mesenchymal stem cells. *Molecules and Cells*.

[37] Lamghari M., Berland S., Laurent A., Huet H., Lopez E. (2001). Bone reactions to nacre injected percutaneously into the vertebrae of sheep. *Biomaterials*.

[38] Xu J., Rao Y., Wu X. (2019). The osteoinductive effect of nano-nacre particles on MC-3T3 E1 preosteoblast through controlled release of water soluble matrix and calciumions. *Dental Materials Journal*.

[39] Willemin A. S., Zhang G., Velot E. (2019). The effect of nacre extract on cord blood-derived endothelial progenitor cells: a natural stimulus to promote angiogenesis?. *Journal of Biomedical Materials Research Part A*.

[40] Silve C., Lopez E., Vidal B. (1992). Nacre initiates biomineralization by human osteoblasts maintained in vitro. *Calcified Tissue International*.

[41] Gerhard E. M., Wang W., Li C. (2017). Design strategies and applications of nacre-based biomaterials. *Acta Biomaterialia*.

[42] Westbroek P., Marin F. (1998). A marriage of bone and nacre. *Nature*.

[43] Atlan G. (1999). Interface between bone and nacre implants in sheep. *Biomaterials*.

[44] Duplat D., Chabadel A., Gallet M. (2007). The in vitro osteoclastic degradation of nacre. *Biomaterials*.

